# Clinical Significance of Methicillin-Resistant *Staphylococcus aureus* Colonization on Hospital Admission: One-Year Infection Risk

**DOI:** 10.1371/journal.pone.0079716

**Published:** 2013-11-20

**Authors:** Jessica P. Ridgway, Lance R. Peterson, Eric C. Brown, Hongyan Du, Courtney Hebert, Richard B. Thomson, Karen L. Kaul, Ari Robicsek

**Affiliations:** 1 Department of Medicine, University of Chicago, Chicago, Illinois, United States of America; 2 Department of Pathology, NorthShore University HealthSystem, Evanston, Illinois, United States of America; 3 Department of Medicine, NorthShore University HealthSystem, Evanston, Illinois, United States of America; 4 The University of Chicago Pritzker School of Medicine, Chicago, Illinois, United States of America; 5 Center for Clinical and Research Informatics, NorthShore University HealthSystem, Evanston, Illinois, United States of America; 6 Department of Biomedical Informatics, The Ohio State University Medical Center, Columbus, Ohio, United States of America; 7 Department of Clinical Analytics, NorthShore University HealthSystem, Evanston, Illinois, United States of America; Arizona State University, United States of America

## Abstract

**Background:**

Methicillin-resistant *Staphylococcus aureus* (MRSA) nasal colonization among inpatients is a well-established risk factor for MRSA infection during the same hospitalization, but the long-term risk of MRSA infection is uncertain. We performed a retrospective cohort study to determine the one-year risk of MRSA infection among inpatients with MRSA-positive nasal polymerase chain reaction (PCR) tests confirmed by positive nasal culture (Group 1), patients with positive nasal PCR but negative nasal culture (Group 2), and patients with negative nasal PCR (Group 3).

**Methodology/Principal Findings:**

Subjects were adults admitted to a four-hospital system between November 1, 2006 and March 31, 2011, comprising 195,255 admissions. Patients underwent nasal swab for MRSA PCR upon admission; if positive, nasal culture for MRSA was performed; if recovered, MRSA was tested for Panton-Valentine Leukocidin (PVL). Outcomes included MRSA-positive clinical culture and skin and soft tissue infection (SSTI). Group 1 patients had a one-year risk of MRSA-positive clinical culture of 8.0% compared with 3.0% for Group 2 patients, and 0.6% for Group 3 patients (p<0.001). In a multivariable model, the hazard ratios for future MRSA-positive clinical culture were 6.52 (95% CI, 5.57 to 7.64) for Group 1 and 3.40 (95% CI, 2.70 to 4.27) for Group 2, compared with Group 3 (p<0.0001). History of MRSA and concurrent MRSA-positive clinical culture were significant risk factors for future MRSA-positive clinical culture. Group 1 patients colonized with PVL-positive MRSA had a one-year risk of MRSA-positive clinical culture of 10.1%, and a one-year risk of MRSA-positive clinical culture or SSTI diagnosis of 21.7%, compared with risks of 7.1% and 12.5%, respectively, for patients colonized with PVL-negative MRSA (p = 0.04, p = 0.005, respectively).

**Conclusions/Significance:**

MRSA nasal colonization is a significant risk factor for future MRSA infection; more so if detected by culture than PCR. Colonization with PVL-positive MRSA is associated with greater risk than PVL-negative MRSA.

## Introduction

Owing to heightened concerns about healthcare-associated infections, hospitals often test asymptomatic inpatients for colonization with methicillin-resistant *Staphylococcus aureus* (MRSA) [1]. These tests regularly uncover previously unknown colonization; indeed, 1.5% of the population of the United States carries this organism [2] and prevalence of MRSA colonization upon hospital admission ranges from 3.9% to 13.6% [1], [3]–[5]. The implications for patients of being found colonized are unclear.

Previous work has suggested that inpatients colonized with MRSA are at risk for MRSA infection during the same hospitalization [1], [6]–[14]. One study found this risk to be 11.1% [7]. Another study showed that of patients colonized with MRSA in the intensive care unit (ICU), 38% went on to develop MRSA bacteremia during their ICU stay [14]. Yet the literature addressing the long-term risk of MRSA infection in MRSA-colonized patients is limited. Previous studies relied on small sample sizes or focused on high-risk patient populations [9], [15]–[19].

Further uncertainties exist. Polymerase Chain Reaction (PCR) has come into wide use as a test method in part because it is thought to have superior sensitivity to culture [20], [21]. However, the accuracy of a PCR assay is hard to define in the absence of a culture-based gold standard. In other words, the clinical significance of being nasally “colonized” with PCR-positive but culture-negative MRSA is unclear. This is important because MRSA detected by PCR often cannot be recovered on culture [22], [23]. Further, not all MRSA isolates are alike. The last decade has seen the emergence of community-associated MRSA, a distinct form of this organism characterized by specific pulsed-field types, unique epidemiology, a predilection for causing skin infections, and a frequent association with Panton-Valentine Leukocidin (PVL), a virulence factor whose clinical significance is a subject of controversy [24]–[27]. Thus, the implications for the patient found to carry MRSA may differ based on the type of test used for detection and the variety of MRSA found.

For several years at our four-hospital healthcare organization, universal testing for MRSA nasal colonization with PCR was performed upon admission, and confirmatory culture was done on all positives. Furthermore, all isolates were tested for the presence of PVL. In this context, we conducted a retrospective cohort study of over 100,000 MRSA-tested patients to determine the influence of MRSA colonization status on one-year risk of MRSA infection.

## Methods

### Ethics Statement

This study was approved by the NorthShore University HealthSystem Institutional Review Board (IRB). Written patient consent was not obtained as a waiver of consent was granted by the IRB.

### Study Overview and Data Collection

This was a retrospective cohort study in which we tracked patients for up to a year following an admission MRSA test to determine the risk of future MRSA-positive clinical culture and physician-diagnosed skin and soft tissue infection (SSTI).

Participants were all patients older than 18 years of age admitted to any of the four hospitals within the NorthShore University HealthSystem (NorthShore) network (formerly Evanston Northwestern Healthcare) between November 1, 2006 and March 31, 2011 who were tested for MRSA nasal colonization. Throughout the study period, a policy of universal MRSA admission testing was in effect for all adults. This program was adopted in an effort to eliminate healthcare-associated MRSA infections in a moderate-incidence setting, and has been previously described [3].

Admission testing was obtained by sampling both anterior nares with double-headed premoistened swabs (BBL CultureSwab; Becton Dickinson, Franklin Lake, NJ). These were processed with the BD-GeneOhm real-time PCR test for MRSA (Becton Dickinson). To confirm the presence of MRSA, samples with positive PCR results underwent broth enrichment and were cultured on colistin-nalidixic acid agar with 5% sheep blood (Remel, Lenexa, KS) and subcultured to MRSA CHROMagar (Becton Dickinson, Franklin Lake, NJ). Colonies underwent Staphaurex latex agglutination test (Murex Biotech, Dartford, United Kingdom) followed by PCR testing for the presence of the *mecA* gene. All nasal cultures positive for MRSA from April 19, 2007 through the end of the study period were further tested for PVL by PCR detection of the *lukF* gene [28].

Patients were classified into three Groups at the time of admission: Group 1 - positive MRSA PCR test with positive confirmatory culture; Group 2 - positive MRSA PCR test but negative confirmatory culture (i.e. positive PCR was of uncertain significance); Group 3 - negative MRSA PCR.

We used the NorthShore Enterprise Data Warehouse (EDW) [29], [30] to obtain data regarding each patient’s characteristics at the time of admission. These included a measure of chronic medical comorbidity, the Charlson comorbidity index [31], and a measure of disease acuity, the Modified Acute Physiology Score (APACHE II score without points for Glasgow Coma Scale) [32].

We followed patients for two outcomes: future MRSA-positive clinical culture and future SSTI. ‘Future MRSA-positive clinical cultures’ were positive non-surveillance cultures collected between 8 and 365 days after the admission surveillance test (i.e. clinical cultures obtained as part of a work-up for infection that grew MRSA). Positive cultures collected 4–7 days after the admission test were all reviewed and then classified as ‘future MRSA-positive clinical cultures’ if they were related to a condition acquired after the admission test (e.g. patient admitted for cardiac surgery developed hospital-acquired pneumonia on day 6 after admission). To identify ‘future SSTI,’ we reviewed all physician-documented (not administrative) diagnoses given to patients between 8 and 365 days following the admission test.

In addition to the above data, we also used the EDW to identify positive MRSA clinical cultures prior to admission (>7 days before the admission test) or concurrent with the admission test (7 days before through 3 days after admission test, and 4–7 days after the admission test if chart review revealed the clinical infection to be present at the time of the admission test). Furthermore, to control for the impact of mupirocin-based decolonization therapy on the risk of MRSA infection (physicians prescribed a five-day decolonization regimen for PCR-positive patients at their own discretion), we determined whether each patient had been treated with mupirocin within 7 days of an admission test.

Two analyses were performed using the data collected. The aim of the first analysis was to determine the one-year risk of future MRSA-positive clinical culture or SSTI, stratified by the following factors: whether a patient’s surveillance test was positive, which test was positive (PCR only or PCR and confirmatory culture), and the PVL status of their MRSA. The aim of the second analysis was to determine the independent influence of the above factors on risk of a subsequent positive clinical culture after controlling for potential confounders.

### Analysis 1: One-Year Infection Risk

**Figure 1:**
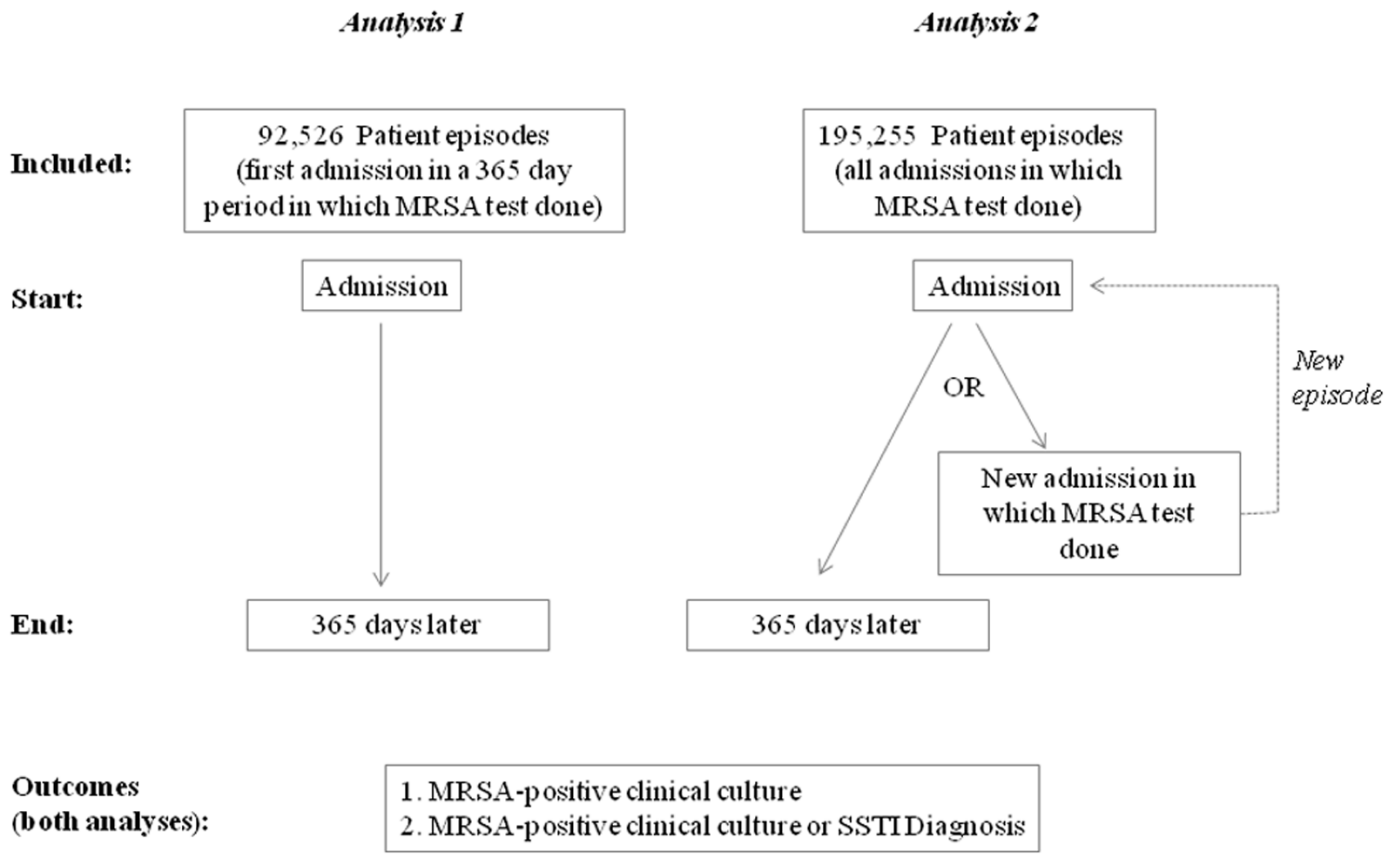
Summary of Analyses. In Analysis 1, patients were followed for 365 days for the outcomes of interest. In Analysis 2, an ‘episode’ was defined as the time from the admission test until the first of these occurrences: a) 365 days elapsed; b) the patient was readmitted and underwent another surveillance test (and thus potentially re-categorized); c) the patient experienced the outcome of interest. Upon readmission, a new episode began and the patient was re-categorized into Group 1, 2, or 3 according to the results of the new nasal swab.

In our first analysis, all patients who underwent admission surveillance testing for MRSA between November 1, 2006 and March 31, 2010 were followed for 365 days for the outcomes of interest (Figure 1). After 365 days elapsed from the initial surveillance test, patients were eligible to be re-entered into the study if they were readmitted and had another admission surveillance test performed.

The primary outcome of interest was future MRSA-positive clinical culture, as defined above. Because of concerns that MRSA infections, especially those caused by community-associated MRSA (i.e. SSTI), are often not cultured, we performed an additional analysis for patients with positive MRSA tests in whom the PVL status was known (Group 1 patients from April 19, 2007 onwards) in which the outcome of interest was MRSA-positive clinical culture or SSTI. Only the first outcome in the 365-day follow-up period was included.

Because some positive clinical cultures from non-invasive sources (e.g. sputum, wound) do not represent true infection, we estimated true infection risk in the following way. We classified each clinical culture according to its source: urine, sputum (including Bronchoalveolar Lavage), SSTI (including swabs from abscesses and wounds), invasive, or other (e.g. samples from sinuses, catheters, chronic ulcers, etc.). We then performed chart review to determine whether cultures represented infection or colonization for 100 random cultures of each non-invasive source Infections were defined in the following way [3]: respiratory tract infection = positive respiratory culture, compatible chest radiograph, and decision to treat with antibiotics; urinary tract infection = positive urine culture and either a decision to treat or growth of more than 100,000 colony-forming units/mL on urine culture plus at least 50 leukocytes per high-power field on urinalysis; SSTI = either decision to treat with antibiotics or decision to drain (i.e. an abscess); “other” infection = decision to treat with antibiotics. We then calculated the true infection proportion for each source, and multiplied the number of positive clinical cultures by this proportion pro-rated by the frequency of cultures from that source to determine the overall true infection rate. To assess severity and impact of MRSA infections, we determined the percentage of patients with a positive clinical culture who died within 14 days of the culture and whether the cause of death was related to infection.

A potential drawback in this kind of retrospective analysis is outcome misclassification because of loss to follow-up (e.g. unrecognized infections because patients received post-admission care outside of our health network). NorthShore represents the dominant healthcare network in its catchment area, with four hospitals and over 100 outpatient practices in close proximity, with universal participation in a single electronic medical record system and use of a single microbiology laboratory, all of which feed into the Enterprise Data Warehouse. Despite this, some patients do receive care at other ambulatory practices and hospitals outside this network. To address this concern, we performed a sensitivity analysis restricted to patient episodes with excellent follow-up, which we defined as those with a NorthShore primary care provider (PCP) and at least one NorthShore healthcare visit within the subsequent year.

We made comparisons using the Chi square test with Stata 11 statistical software package (StataCorp, College Station, TX).

### Analysis 2: Association between Nasal Colonization and Future Clinical Culture

The second analysis determined the independent influence of nasal colonization status on risk of future MRSA-positive clinical culture in a multivariable model. For this analysis, all inpatient encounters during the study period were included. Unlike the previous analysis, in Analysis 2 patients were not necessarily followed for an entire year (Figure 1). Rather, an ‘episode’ was defined as the time from the admission test until the first of these occurrences: a) 365 days elapsed; b) the patient was readmitted and underwent another surveillance test (and thus potentially re-categorized); c) the patient experienced the outcome of interest. Upon readmission, a new episode began and the patient was re-categorized into Group 1, 2, or 3 according to the results of the new nasal swab (see above). For Analysis 2, we chose to reclassify patients if their nasal colonization status changed so that we could characterize the association between colonization status and infection risk as accurately as possible. (We did not do this in Analysis 1. There we adopted the perspective of a clinician considering their patient’s one-year risk of infection without knowledge of future test results.) Outcomes of interest in Analysis 2 were as in Analysis 1.

Time-to-event analysis was used to determine the association between nasal colonization (i.e. Group 1, 2 or 3) and the outcomes of interest. Time-to-event difference among groups was depicted using Kaplan-Meier curves and log-rank test, and univariate and multivariable Cox regression modeling were performed [33], [34]. The proportionality assumption was assessed and fulfilled for all variables analyzed with this method. As some patients experienced repeated tests and/or recurrent events, a conditional Cox regression model accounting for within-subject correlation was also used [35]. The multivariable models included the following factors to control for underlying differences between patients in the different colonization groups: age, sex, ethnicity, long-term care facility residence, prior history of belonging to Group 1, 2, or 3, hospitalization in the prior year, surgery within the next 7 days, pressure ulcer in the prior year, weight greater than 80 kilograms, and Charlson comorbidity index. A subgroup analysis was performed including Modified Acute Physiology Score as a covariate for the episodes in which it was available (66% of episodes; the remainder of patients did not have all laboratory tests needed for score calculation available within the first 24 hours of admission). In a separate multivariable model for only Group 1 patients (the only patients in whom PVL status was known), prescription of mupirocin and PVL results were included in addition to the above variables. We also performed a sensitivity analysis restricting our analysis to patients with excellent follow-up, as defined previously. Statistical analyses were performed in R 2.15.3 using the RMS and survival packages [36], [37] and SAS 9.2 (SAS Institute, Cary, NC).

## Results

### Analysis 1: One-Year Infection Risk

There were 92,526 patient encounters included in Analysis 1, of whom 80,191 were unique patients (Table 1). The one-year risk of future MRSA-positive clinical culture was higher for Group 1 patients (8.0%) than for patients in Group 2 (3.0%) or Group 3 (0.6%), (p<0.001) (Table 2). Risk was higher for patients who had past or concurrent MRSA-positive clinical cultures than for those who were only colonized. Risk was equivalent for all three nasal colonization Groups for patients with a MRSA-positive clinical culture at the time of admission (i.e. concurrent culture) (p = 0.94). In the sensitivity analysis, the risk of future positive clinical culture for the subset of patients with excellent follow-up was similar to the risk found in the general population (Table 3), suggesting limited misclassification.

**Table 1:** Baseline Characteristics of Patient Episodes in Analysis 1.

Baseline Characteristic	Group 1 (PCR+/NC+) (n = 2631)	Group 2 (PCR+/NC−) (n = 1234)	Group 3 (PCR−) (n = 88661)
Age, median (IQR), y	75 (56–85)	63 (46–80)	58 (39–76)
Female, sex	1499 (57.0)	747 (60.5)	56671 (63.9)
African American	221 (8.4)	126 (10.2)	6432 (7.3)
American Indian	3 (0.1)	5 (0.4)	289 (0.3)
Asian/Pacific Islander	49 (1.9)	41 (3.3)	2641 (3.0)
Caucasian	2102 (80.0)	861 (69.9)	65764 (74.3)
Hispanic	31 (1.2)	39 (3.2)	3542 (4.0)
Other	221 (8.4)	160 (13.0)	9899 (11.2)
Long-term care facility residence	468 (17.8)	119 (16.1)	2280 (2.6)
Hospitalization in prior year	556 (21.1)	168 (13.6)	6836 (7.7)
Pressure ulcer in prior year	549 (20.9)	132 (10.7)	3652 (4.1)
Weight >80 kg	1109 (42.2)	549 (44.5)	39063 (44.1)
Surgical patient (surgery within next7 days)	315 (12.0)	317 (25.7)	21009 (23.7)
Charlson comorbidity index, median(IQR, no. with data available)	1 (0–2, n = 2529)	0 (0–1, n = 1185)	0 (0–0, n = 86509)
Modified APS, median (IQR, no. withdata available)	11 (8–14, n = 1959)	10 (7–13, n = 801)	9 (6–12, n = 48818)

Abbreviations: IQR, interquartile range; NC, nasal culture; PCR, polymerase chain reaction; APS, Acute Physiology Score.

Data are reported as No. (%) unless otherwise indicated.

**Table 2:** One-Year Risk of MRSA-positive Clinical Culture Stratified by Group and History of MRSA.

	Group 1(PCR+/NC+)					Group 2(PCR+/NC−)					Group 3(PCR−)					Overall PvalueAmongGroups
	All Group 1	Past MRSA*	ConcurrentMRSA	No past orconcurrent MRSA	P value	AllGroup 2	PastMRSA*	ConcurrentMRSA	No past orconcurrent MRSA	P value	AllGroup 3	PastMRSA*	ConcurrentMRSA	No past orconcurrent MRSA	P value	
**One-year risk of** **MRSA-positive** **clinical culture**	**211/2631** **(8**.**0)**					**37/1234** **(3**.**0)**					**523/8866** **(0**.**6)**					**<0**.**001**
**One-year risk of** **MRSA-positive** **clinical culture**		58/406 (14.3)	43/239 (18.0)	110/1986 (5.5)	<0.001		3/73 (4.1)	8/49 (16.3)	26/1112 (2.3)	<0.001		60/1053 (5.7)	30/162 (18.5)	433/87446 (0.5)	<0.001	

Abbreviations: NC, nasal culture; PCR, polymerase chain reaction; SSTI, skin and soft tissue infection.

Data are reported as no. episodes with outcome/total no. episodes (%).

* Includes patients with a history of MRSA clinical culture or a history of Group 1 and excludes patients with concurrent clinical cultures.

**Table 3:** One-Year Risk of MRSA-positive Clinical Culture Among Patients with Excellent Follow-up Stratified by Group and History of MRSA.

	Group 1 (PCR+/NC+)					Group 2 (PCR+/NC−)					Group 3 (PCR−)					Overall P value among groups
	AllGroup 1	PastMRSA*	ConcurrentMRSA	No past orconcurrent MRSA	P value	AllGroup 2	PastMRSA*	ConcurrentMRSA	No past orconcurrent MRSA	P value	AllGroup 3	PastMRSA*	ConcurrentMRSA	No past orconcurrent MRSA	P value	
**One-year risk of MRSA-positive clinical culture among patients with excellent follow-up**	**84/829 (10.1)**					**12/427 (2.8)**					**228/35285 (0.6)**					**<0.001**
**One-year risk of MRSA-positive clinical culture among patients with excellent follow-up**		27/159 (17.0)	19/81 (23.5)	38/589 (6.5)	<0.001		1/26 (3.8)	3/15 (20.0)	8/386 (2.1)	<0.001		31/451 (6.9)	13/59 (22.0)	184/34775 (0.5)	<0.001	

Abbreviations: NC, nasal culture; PCR, polymerase chain reaction.

Data are reported as no. episodes with outcome/total no. episodes (%).

* Includes patients with a history of MRSA clinical culture or a history of Group 1 and excludes patient episodes with concurrent clinical cultures.

Patients in Group 1 with PVL-positive MRSA colonization had a significantly higher risk of future MRSA-positive clinical culture and ‘future MRSA-positive clinical culture or SSTI diagnosis’ than those with PVL-negative MRSA (Table 4).

**Table 4:** One-year risk of MRSA-positive Clinical Culture and ‘MRSA-positive Clinical Culture or SSTI Diagnosis’ in Group 1 Patients According to PVL status.

	PVL positive	PVL negative	P value
**One-year risk of MRSA-positive clinical** **culture**	40/396 (10.1)	125/1769 (7.1)	0.04
**One-year risk of ‘MRSA-positive clinical** **culture or SSTI diagnosis’**	86/396 (21.7)	221/1769 (12.5)	0.005

Abbreviations: PVL, Panton-Valentine Leukocidin; SSTI, skin and soft tissue infection.

Data are reported as no. episodes with outcome/total no. episodes (%).

Mupirocin therapy was given in 77% of Group 1, 80% of Group 2, and 4% of Group 3 encounters. The one-year risk of MRSA clinical culture in Group 1 patients was 8.3% for those prescribed mupirocin and 7.4% for those who were not prescribed mupirocin (p = 0.54).

Of the 400 MRSA-positive clinical cultures that underwent chart review, 74% of urine cultures, 76% of sputum cultures, 95% of SSTI cultures, and 79% of other cultures represented true infections. Future MRSA-positive clinical cultures in the study included 12% invasive, 13% urine, 11% sputum, 45% SSTI, and 19% other cultures, suggesting a total ‘true infection’ rate of 7.0%, 2.6% and 0.5% in Groups 1, 2, and 3 respectively.

There were 773 episodes associated with a positive clinical culture within the subsequent year. Of these, 54 (7%) died within 14 days of the clinical culture. In 41 patients (76% of those who died), the cause of death was an infection.

### Analysis 2: Association between Nasal Colonization and Future Clinical Culture

Over the study period, there were 219,244 admissions of which 195,255 (89.1%) were MRSA-tested, representing 109,857 unique patients. (Analysis 2 included more unique patients than Analysis 1 since Analysis 1 was limited to patients for whom an entire year of follow-up was available). There were 10,530 positive PCR tests; 6964 were positive by confirmatory culture (66.1%, Group 1) and in the remaining 3566 MRSA confirmatory cultures were negative (33.9%, Group 2) (Table 5).

**Table 5:** Baseline Characteristics of Patient Episodes in Analysis 2.

Baseline Characteristic	Group 1 (PCR+/NC+) (n = 6964)	Group 2 (PCR+/NC−) (n = 3566)	Group 3 (PCR−) (n = 184725)
Age, median (IQR), y	78 (60–86)	71 (53–83)	63 (45–79)
Female, sex	3670 (52.7)	1961 (55.0)	113549 (61.5)
African American	621 (8.9)	345 (9.7)	14479 (7.8)
American Indian	8 (0.1)	7 (0.2)	570 (0.3)
Asian/Pacific Islander	119 (1.7)	75 (2.1)	5036 (2.7)
Caucasian	5619 (80.8)	2690 (75.5)	138448 (75.0)
Hispanic	79 (1.1)	96 (2.7)	6666 (3.6)
Other	512 (7.4)	350 (9.8)	19368 (10.5)
Long-term care facility residence	2082 (29.9)	758 (21.3)	11254 (6.1)
Hospitalization in prior year	3930 (56.4)	1762 (49.4)	50075 (27.1)
Pressure ulcer in prior year	2223 (31.9)	849 (23.8)	16058 (8.7)
Weight >80 kg	3077 (44.2)	1689 (47.4)	83743 (45.3)
Surgical patient (surgery within next 7 days)	764 (11.0)	732 (20.6)	42533 (23.0)
Charlson comorbidity index, median(IQR, no. with data available)	2 (0–4, n = 6610)	1 (0–3, n = 3218)	0 (0–1, n = 178001)
Modified APS, median (IQR, no. withdata available)	12 (9–16, n = 5667)	11 (8–15, n = 2503)	10 (7–13, n = 119759)

Abbreviations: IQR, interquartile range; NC, nasal culture; PCR, polymerase chain reaction; APS, Acute Physiology Score.

Data are reported as No. (%) unless otherwise indicated.

In the multivariable model, hazard ratios for future MRSA-positive clinical culture using Group 3 (PCR negative) as the reference group were 6.52 (95% CI, 5.57–7.64; p<0.0001) for Group 1 (PCR positive and confirmatory culture positive) and 3.40 (95% CI, 2.70–4.27; p<0.0001) for Group 2 (PCR positive but confirmatory culture negative). The conditional Cox regression model accounting for recurrent events yielded similar results (data not shown). In subgroup analyses, hazard ratios were similar in the subset of patients with excellent follow-up and in patients for whom a Modified Acute Physiology Score was available (Table 6).

**Table 6:** Hazard Ratios for Association Between MRSA Nasal Colonization and Future MRSA-positive Clinical Culture in Multivariable Model Subgroup Analyses.

	Group 1 (PCR+/NC+)*HR (95% CI)	P value	Group 2 (PCR+/NC−)*HR (95% CI)	P value
**All Patients**	6.52 (5.57 to 7.64)	<0.0001	3.40 (2.70 to 4.27)	<0.0001
**Patients with Excellent Follow-up**	6.00 (4.25 to 7.78)	<0.0001	4.02 (2.82 to 5.72)	<0.0001
**Patients with Modified Acute Physiology Score Available**	5.95 (5.02 to 7.06)	<0.0001	2.67 (2.03 to 3.52)	<0.0001

Abbreviations: NC, nasal culture; PCR, polymerase chain reaction; HR, hazard ratio.

* Reference is Group 3 (PCR−).

**Figure 2:**
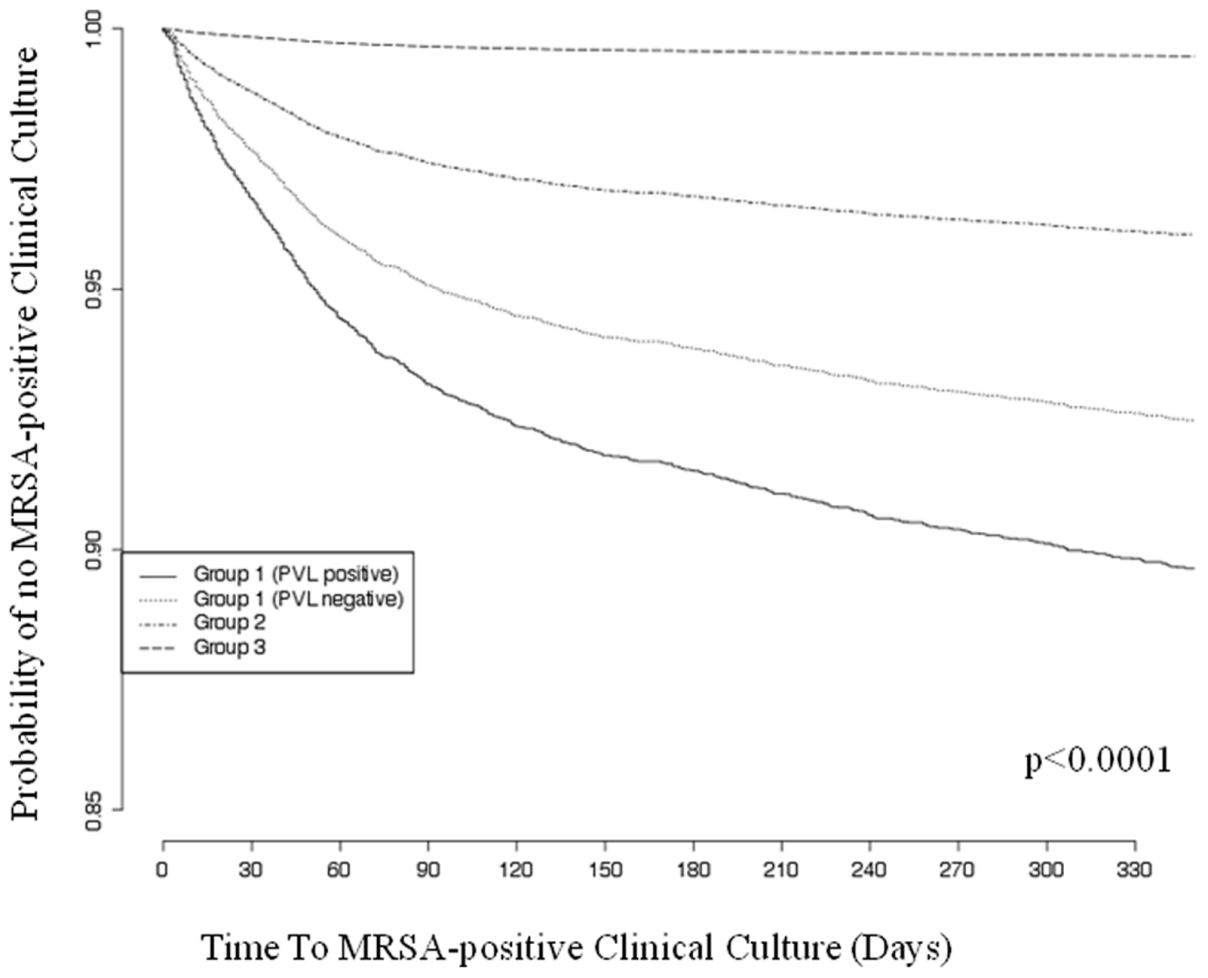
Kaplan Meier Curve of MRSA-positive Clinical Culture-free Survival Stratified by Group.

In the multivariable model that only included Group 1 patients, individuals colonized with PVL-positive MRSA had an increased risk of future MRSA-positive clinical culture compared with those colonized with PVL-negative MRSA (HR 1.56, 95% CI, 1.02–2.39; p = 0.04). They also had an increased risk of the combined outcome ‘future MRSA-positive clinical culture or SSTI diagnosis’ (HR 2.51, 95% CI, 1.95–3.22; p<0.0001). There was no significant difference in the risk of future clinical culture for Group 1 patients prescribed mupirocin compared to Group 1 patients not prescribed mupirocin (HR 1.28, 95% CI, 0.82–2.01; p = 0.28). Figure 2 shows future culture-free survival over time by colonization Group and PVL status.

## Discussion

Legislative mandates and concerns regarding healthcare-associated infections have led many healthcare systems – including all hospitals belonging to the United States Veterans Health Administration – to adopt a policy of universal admission testing for MRSA [1], [4], [38]. Thousands of patients are annually identified as MRSA-colonized, but limited evidence has been available to counsel patients regarding their risk of infection. In a population of more than 100,000 adult patients evaluated for MRSA as a part of a universal surveillance initiative, we characterized infection risk associated with different colonization states.

The one-year risk of having a MRSA-positive clinical culture in patients whose PCR test was positive (Group 1 or 2) was 6.4%, compared to 0.6% if the test was negative. Within the positive-test population, there was considerable variation in that risk, depending on whether the confirmatory culture was positive (8.0% if so, 3.0% if not), whether the patient had a history of a MRSA-positive culture, and whether the MRSA was PVL-positive (10.1% if so, 7.1% if not). Patients with past history of MRSA-positive culture, regardless of Group, were more likely to have a future MRSA-positive clinical culture than those with a positive surveillance PCR test (7.9% vs. 6.4%, p = 0.05). No matter the nasal colonization status, patients who presented to the hospital with a culture-positive MRSA infection stood approximately an 18% chance of a future positive culture.

The 7.0% risk of ‘true’ future MRSA infection in culture-proven MRSA-colonized (Group 1) patients in this study was lower than the 10–33% risk estimated in prior studies [9], [15]–[19]. This difference may be due to the composition of our study population. While other studies were largely conducted at tertiary referral centers and identified study participants via targeted screening of high-risk patients or based on positive clinical cultures with MRSA [15]–[17], we describe outcomes in a population of nearly universally-tested inpatients at four general hospitals in the Midwestern United States.

We found that patients in Group 2 (those with a positive PCR but negative nasal culture) had lower risk of MRSA infection than Group 1 patients. This may indicate that a low density of viable organisms (accounting for negative culture but positive PCR) predicts low risk of eventual infection. Alternatively, Group 2 patients may comprise a mix of patients with true colonization who have the same risk of MRSA-positive culture as Group 1 patients (8%), and patients with false positive tests who have the same risk as Group 3 patients (0.6%). If this is the case, our observed one-year risk of 3% in Group 2 would suggest that roughly two thirds of Group 2 patients were not actually colonized. As Group 2 patients accounted for 33.9% of all positive PCR tests, as many as 20% of positive PCR tests would have been false positives. Stated differently, much of the apparently greater sensitivity of PCR over culture may actually represent false positive tests. Potential causes for false positives include the presence of *S. aureus* with a staphylococcal cassette chromosome element lacking the *mecA* gene (*mecA* dropout) or a large inoculum of methicillin-sensitive *S. aureus* [21], [39].

PVL is a cytotoxin produced by some strains of *S. aureus* that is epidemiologically associated with SSTI and necrotizing pneumonia [40]–[42] and is closely associated with community-associated MRSA (pulsed-field type USA300) in our population [25], [28]. The clinical significance of this virulence factor is disputed; one group has reported increased odds of bacteremia in ICU patients colonized with PVL-positive as opposed to PVL-negative MRSA [43], but others have not found PVL to be associated with worse clinical outcomes [44]–[46]. We found that colonization with PVL-positive MRSA was associated with increased risk of future MRSA-positive clinical culture compared with colonization with PVL-negative MRSA (HR 1.56). This difference was especially pronounced when uncultured SSTI were included in the analysis.

Our study has limitations. The nares are not the only site of MRSA colonization, and patients who had a negative nasal swab for MRSA may have been colonized at other sites. With the large number of patient encounters included in our study, there was almost certainly loss to follow-up. However, a sensitivity analysis in which we only considered patients with excellent follow-up yielded very similar results. Our primary outcome was future MRSA-positive clinical culture, rather than MRSA infection. While a random record audit found that most positive cultures represented true infections, our results may still overestimate MRSA infection risk. On the other hand, the potential for us to have missed infections without a positive culture or SSTI diagnosis may have caused some risk underestimation. This study included patients from only four hospitals, but the risk of MRSA infection among patients with MRSA nasal colonization was not significantly different between the four hospitals (data not shown), suggesting generalizability.

We have characterized the risk associated with different MRSA surveillance test results in a large universally-tested patient population. These data can be used to improve disease transmission and economic models [47], [48], inform infection control and laboratory planning, and guide the process of counseling patients about their risks.
